# Unraveling plant adaptation to nitrogen limitation from enzyme stoichiometry aspect in Karst soils: a case study of *Rhododendron Pudingense*

**DOI:** 10.3389/fpls.2023.1267759

**Published:** 2023-11-30

**Authors:** Haodong Wang, Baoxian Huang, Hongjiu Zhao, Xiaoyong Dai, Meng Chen, Fangjun Ding, Peng Wu, Lei Hao, Rui Yang, Congjun Yuan

**Affiliations:** ^1^ College of Forestry, Guizhou University, Guiyang, China; ^2^ Guizhou Academy of Forestry, Guiyang, China; ^3^ National Positioning Observation and Research Station of Guizhou Libo Karst Forest Ecosystem, Libo, Guizhou, China; ^4^ Guizhou Provincial Institute of Biology, Guiyang, Guizhou, China

**Keywords:** Karst microhabitat, enzyme stoichiometry, *Rhododendron pudingense*, nutrient allocation, N limitation

## Abstract

Enzyme stoichiometry can reflect the resource limitation of soil microbial metabolism, and research on the relationships between plants and resource limitation in Karst Microhabitats is scarcely investigated. To clarify the extracellular enzyme stoichiometry characteristics in soil across different karst microhabitats and how the *Rhododendron pudingense* adapts to nutrient restrictions, plot investigation experiments were set up in Zhenning County, Qinglong County, and Wangmo County of Guizhou Province which included total three karst microhabitats, *i.e.*, soil surface (SS), rock gully (RG), and rock surface (RS), by analyzing he rhizosphere soil nutrient, extracellular enzyme activity, and nutrient content of *R. pudingense*. The findings indicated that all karst microenvironments experienced varying levels of nitrogen (N) limitation, with the order of N limitation being as follows: SS > RG > RS. Notably, there were significant discrepancies in N content among different plant organs (*p*< 0.05), with the sequence of N content as follows: leaf > stem > root. However, no significant differences were observed in nutrient content within the same organ across different microenvironments (*p* > 0.05). A noteworthy discovery was the significant allometric growth relationship between C-P in various organs (*p*< 0.05), while roots and stems exhibited a significant allometric growth relationship between N-P (*p*< 0.05). The study highlighted the substantial impact of Total Nitrogen (TN) and N-acquiring enzymes (NAE) on nutrient allocation within the components of R. pudingense. Overall, the research demonstrated that N was the primary limiting factor in the study area’s soil, and R. pudingense’s nutrient allocation strategy was closely associated with N limitations in the karst microenvironment. Specifically, the plant prioritized allocating its limited N resources to its leaves, ensuring its survival. This investigation provided valuable insights into how plants adapt to nutrient restrictions and offered a deeper understanding of soil-plant interactions in karst ecosystems.

## 1 Introduction

The karst landforms in Southwest China are concentrated and widely distributed (Song, 2015), exhibiting high habitat heterogeneity, vast ecological space, rich biological resources (Xi et al., 2011), fragility and harshness. Zhu (1993) divided six kinds of karst microhabitats, such as rock surface, rock gully, rock trough, rock hole, rock crevice and soil surface, according to the morphology of rock-soil preservation. The study of microhabitat-scale is conducive to our in-depth understanding of the plant-environment interactions in karst regions. In 2020, a previously unidentified plant species within the *Ericaceae* family, *Rhododendron pudingense*, was first observed in Puding County, Anshun City, Guizhou Province, China. This newly discovered species is characterized by its pink blossoms and has a predilection for the higher regions of karst mountains (Dai et al., 2020). It has since been found in Zhenning County, Qinglong County, Wangmo County, Kaiyang County and other places in Guizhou Province, and is an endemic species to Guizhou. Interestingly, while native *Rhododendron* plants mainly grow in acidic soils (Su et al., 2020), this species thrives only in weak alkaline soil in karst areas, highlighting its unique adaptation mechanisms that require further study. Notably, study of Rhododendron pudingense is significant due to its ecological importance, unique characteristics, and potential contributions to conservation, medicine, and horticulture (Yuan et al., 2023). This species plays a role in supporting biodiversity within its habitat, and its distinctive features add to our understanding of plant diversity. Investigating its ecological function sheds light on its interactions with other organisms, while exploring its adaptations to nutrient limitation can provide insights into plant-soil relationships (Sinsabaugh et al., 2008).

It’s noteworthy that soil enzymes play a critical role as biological catalysts in ecosystem function, promoting soil material cycling and energy flow (Wang et al., 2016). They are classified into extracellular enzymes, intracellular enzymes, and free enzymes, and are mainly generated through the decomposition of animal and plant remnants, exudates of plant roots, and microbial activity in soil (Cao et al., 2003). Microbes secrete extracellular enzymes that are indispensable to the degradation of soil organic matter (Fan et al., 2018). Moreover, these enzymes participate in almost all chemical reactions in the soil, thereby stimulating soil organic matter decomposition and nutrient cycling (Asmar et al., 1994; Leff et al., 2015). However, when a soil microbe’s internal elemental balancing mechanism and environmental resource supply and demand are imbalanced, it might lead to microbial nutrient limitations. When that happens, microbes compete with plants for nutrients, making microbial nutrient constraints related to plant nutrient deficiencies (Inselsbacher et al., 2010). Therefore, it is highly likely that nutrient limitation in microorganisms is closely linked to nutrient limitation in plants. Researchers have paid greater attention to microbial nutrient limitations recently (Cui et al., 2021; Wang et al., 2023).

In addition, enzyme stoichiometry has been used to explore the characteristics of soil microbial nutrient restriction in karst regions, mainly aiming at different land use modes (Sun et al., 2021) and different rock desertification degrees (Sun et al., 2022). However, it is not clear whether there are differences in microbial nutrient restriction among different karst microhabitats in the same succession stage and similar forest vegetation types. Assuming the existence of nutrient limitation in the soil of the study area, it remains unknown as to what nutrient allocation strategy the native plant *R. pudingense* would adopt under such a nutrient limitation regime. Ecological stoichiometry theory holds that there is a dynamic balance between energy and chemical elements such as carbon (C), nitrogen (N), and phosphorus (P) in ecosystems (Elser et al., 2000; Cheng et al., 2010). Some researchers contend that multiple extracellular enzymes related to the microbial acquisition of C, N, and P elements like *β*-1,4-glucosidase (BG), *β-*1,4-N-acetylglucosaminidase (NAG), L-leucine amino peptidase (LAP) and alkaline phosphatase. (AP), also exist with ecological stoichiometry relationships. By gauging the percentage of these extracellular enzyme activities, we can assess the degree of microbial need for C, N, and P elements and derive the notion of soil enzyme stoichiometry (Schimel and Weintraub, 2003; Hill et al., 2006; Moorhead and Sinsabaugh, 2006). Ecological enzyme stoichiometry is also commonly used to appraise the characteristics of nutrient metabolism inhibition in soils by microbes (Sinsabaugh et al., 2009; Cui et al., 2021). The current studies mainly focus on different geographic scales, including global (Sinsabaugh et al., 2008), river basin (Hill et al., 2012), and north-south transect scales of eastern China (Xu et al., 2017). However, these conclusions may not be universally applicable.

C, N and P are essential nutritional elements required for plant growth (Sardans et al., 2012). They not only compose the cell metabolism, proteins and genetic material (Cui et al., 2018), but also play an important role in regulating various physiological functions of plants (Rong et al., 2012; Huang et al., 2019). Researchers use chemical stoichiometry ratios of C, N and P to identify the nutrient limitation status of plants (Tessier and Raynal, 2003; Gallardo and Covelo, 2005) and nutrient allocation strategies (Chen et al., 2021). They have consistently concluded that the nutrient content and proportional relationship of a single organ cannot directly reflect the situation of other organs or the entire plant, making it important not to ignore the interaction between different organs (Zhang et al., 2020; Zhao et al., 2021). The study on chemical stoichiometry of roots, stems and leaves can help us understand the nutrient allocation strategy during the plant growth process (Yao et al., 2023), as well as reveal how plants utilize resources (Wang X. et al., 2015). In the research conducted by Zhao et al. (2014), they studied the nutrient contents and chemical stoichiometry ratios of the fine roots, stems and leaves of *Larix principis-rupprechtii* plantation in North China. Their findings showed that the C, N and P chemical stoichiometry ratios of fine roots were relatively stable and not affected by the growth season. Chen et al. (2016) discovered that the nutritional elements in different organs of *Cunninghamia lanceolata* are mobile and interact with each other based on their analysis of inter-organ chemical stoichiometric characteristics. Therefore, using ecological stoichiometry can better understand how elements couple among different organs of plants (Chen et al., 2022), helping to reveal how plants cope with potential resource limitations in karst regions.

Based on above consideration, the study’s objective is to investigate nutrient allocation strategies in the native plant species *R. pudingense* within karst regions of Southwest China, with a specific focus on the relationship between microbial nutrient limitation and plant nutrient allocation. It aims to assess potential differences in microbial nutrient restriction among different karst microhabitats and their impact on R. pudingense’s adaptation to nutrient-limited soils. Additionally, the study explores ecological enzyme stoichiometry relationships to understand soil nutrient metabolism inhibition and examines how chemical stoichiometry ratios of carbon (C), nitrogen (N), and phosphorus (P) in different plant organs (roots, stems, leaves) of *R. pudingense* can elucidate its nutrient allocation strategies, ultimately shedding light on how this plant copes with resource limitations in karst environments. Findings from this research will contribute to providing theoretical evidence for the protection of unique germplasm resources and innovative utilization of *R. pudingense* in karst rocky habitats, enrich the theories of adaptability and ecological restoration of karst plants, and hold significant implications for the recovery of karst vegetation.

## 2 Materials and methods

### 2.1 Study area and Karst microhabitat division

The study area is located in Xinfa Village of Zhennin County (ZN), Hama Community of Qinglong County (QL), and Heidong of Wangmo County (WM) in Guizhou Province, China (**Figure S1**). The three regions are situated between 105°1′ to 106°49′E and 24°53′ to 26°11′N in the southwestern Guizhou Province, characterized by a high northwest terrain and low southeast terrain that belongs to the southwestern karst plateau zone at an elevation of approximately 1200–1450 m above sea level. The region has a subtropical monsoon humid climate with the following characteristics: no severe cold in winter, no scorching heat in summer, rainy season coinciding with hot weather, and warm and moist during the festival season. The annual average temperature is about 14.2–19.7 °C and the annual average rainfall is around 1000–1600 mm. The soil on the limestone mountain is black lime soil, and the forest community plant species are primarily composed of *Platycarya longi*p*es*, *Carpinus pubescens*, *R. pudingense*, etc. (**Table 1**). Based on the research results of Yuan et al. (2023), this study divided the karst microhabitats and selected the most typical microhabitats with *R. pudingense*, namely rock surface (RS), rock gully (RG), and soil surface (SS) within the plot, according to the criteria shown in **Table S1**.

**Table 1:** Basic information on forest community in the study area.

Region	ZN	QL	WM
Elevation	1394.3	1421.2	1220.9
Slope (°)	50	50	50
Arborous layer dominant species	*Platycarya strobilacea*	*Platycarya strobilacea*	*Platycarya strobilacea*
Mean diameter at breast height (cm)	12.4	7.0	6.2
Arborous layer mean height (m)	10.0	7.2	7.9
Arborous layer density (plant/hm^2^)	350	2983	1025
Canopy density (%)	35	85	55
Mean ground diameter of *R. pudingense* (cm)	1.9	1.4	1.6
Mean height of *R. pudingense* (m)	1.8	1.7	1.7

### 2.2 Sample collection

Soil samples were collected from three regions, Zhenning County (ZN), Qinglong County (QL), and Wangmo County (WM), respectively, from July 28th to August 1st, 2022. Three plots (20 m x 10 m) were established in each region with an interval of at least 20 m between neighboring ones. The geographical coordinates, elevation, slope, slope direction, and other related factors were recorded for each plot. Sampling was conducted based on the microhabitat types of *R. pudingense* within sample plots, and information such as underground diameter, plant height, and ground cover of the sampled plants was noted. At least one representative *R. pudingense* plant per microhabitat category was selected for sampling per plot. Samples were taken from healthy plants with similar underground diameters and plant heights, mature leaves were selected for leaf samples, young branches were selected for stem samples, while fine roots and lateral roots were selected for root samples. Around 200g of each sample was stored in self-sealing bags and labeled before being placed in a foam box filled with ice immediately after collection. A total of 27 soil samples were collected from the three regions. After the rhizosphere soil was collected, put it into a foam box with ice packs, about 50g. Brought it back to the laboratory and stored in the refrigerator at –80 °C for the determination of soil nutrients and enzyme activities after the sampling.

### 2.3 Determination of soil physical and chemical properties and enzyme activities

The plant samples are subjected to 105°C drying for 10 minutes in the laboratory after being collected. Next, they are dried at 70°C until a constant weight is reached before being crushed and sieved through a 100-mesh sieve for use as test samples. The soil organic carbon content (SOC) was determined using the potassium dichromate volumetric method, while the total nitrogen content (TN) was determined using the Kjeldahl method and the total phosphorus content (TP) was determined using molybdenum-antimony resistance colorimetry. This study employed enzyme-linked immunosorbent sandwich assays to determine four types of extracellular enzyme activity, including C-acquiring enzyme: β-1,4-glucosidase (BG); N-acquiring enzymes: β-1,4-N-acetylglucosaminidase (NAG) and L-leucine aminopeptidase (LAP); and P-acquiring enzyme: alkaline phosphatase (AP). Different organs’ C content was determined by the potassium dichromate volume method, N content was determined by the Kjeldahl nitrogen determination method, and P content was determined by the molybdenum-antimony anti-colorimetric method.

### 2.4 Data analysis

All extracellular enzymes were logarithmically transformed. The soil extracellular enzyme C:N was expressed as ln(*BG*)/ln(*NAG* + *LA*P), the soil extracellular enzyme C:P was expressed as ln(*BG*)/ln(*A*P), and the soil extracellular enzyme N:P was expressed as ln(*NAG* + *LA*P)/ln(*A*P). The vector characteristics of soil extracellular enzyme activity were calculated according to Moorhead et al. (2016). The calculation formula is as follows:

The vector length reflects the degree of C limitation, with a longer vector indicating stronger C limitation on microbes. The angle of the vector reflects the degree of nitrogen and phosphorus limitation, where an angle greater than 45° indicates P limitation, and an angle less than 45° indicates N limitation. Moreover, this type of limitation becomes stronger as it deviates further from the 45° angle (Moorhead et al., 2016; Cui et al., 2021).

The allometric growth equation was employed to analyze the relationship between C, N, and P in different organs. After the logarithmic transformation of the N and P contents in each organ, the following formula was utilized to conduct calculations and analysis: In the equation (Eq. 3), *x* and *y* represent the contents of C, N and P, *m* is the allometric growth index, which refers to the slope of the allometric growth equation, and *n* is the allometric growth normalization constant, corresponding to the intercept of the equation. All data processing and analysis were completed using Microsoft Excel 2016 and IBM SPSS 26.0. When the enzyme activity data did not conform to a normal distribution, logarithmic transformation was performed to achieve normal distribution before subsequent data analysis. Two-way ANOVA was used to test the significant differences (*p<* 0.05) in soil nutrients, stoichiometry ratios, as well as nutrient distribution across different microhabitats and regions, and to determine the significant differences (*p<* 0.05) in nutrient allocation among different organs within each microhabitat. Pearson correlation analysis was utilized to explore the relationship between soil nutrients and enzyme activities, while Mantel tests were conducted to investigate the correlation between organ nutrient distribution and environmental factors, with Euclidean distance matrix calculation for environmental variable distances and Bray-Curtis distance matrix calculation for organ nutrient distribution distances. Mental test analysis was performed on Tutools platform, a free online data analysis website (http://www.cloudtutu.com). Redundancy analysis was employed to explore the relationships between soil nutrients, enzyme activities, stoichiometry ratios, and organ nutrient characteristics. Canoco 5 was used to draw the figures, while Origin 21.0 was used to draw the bar graph and allometric growth model, and the graphical abstract was created using Adobe Illustrator 2021.


(Eq. 1) $$\text{Vector length }=\sqrt{{\left[\text{ln}\left(BG\right)/\text{ln}\left(NAG+LAP\right)\right]}^{2}+{\left[\ln\left(BG\right)/\text{ln}\left(AP\right)\right]}^{2}}$$

(Eq. 2) Vector angle=Degress{ATAN2[ln(BG)/ln(AP),ln(BG)/ln(NAG+LAP)]}

(Eq. 3) lnx=mlny+n

## 3 Results

### 3.1 Soil C, N and P contents in different karst microhabitats

**Table S2** presented data indicating that while QL soil TN and TP were significantly different in RS and RG compared to SS (p< 0.05), there were no significant differences in the nutrient contents of ZN and QL among the various niches (p > 0.05). Moreover, the soil SOC and TN contents in WM shown significant differences among different niches (p< 0.05). AN content of SS and RS exhibited a significant difference (p< 0.05), while SAP content of RS differed significantly from the other two niches (p< 0.05). The rest of the indices did not present any significant difference (p > 0.05). According to the **Table S3**, niche had a notable influence on each index (p< 0.05), and interregional effects were significant on soil C, N, and P contents (p< 0.05). With the exception of soil C:N, the interaction between niche and region remarkably affected soil nutrient content (p< 0.05). Moreover, the nutrient content of SS was lower, while the nutrient contents of QL and WM were higher than those of ZN.

### 3.2 Extracellular enzyme activity in different karst microhabitats

**Figure 1:**
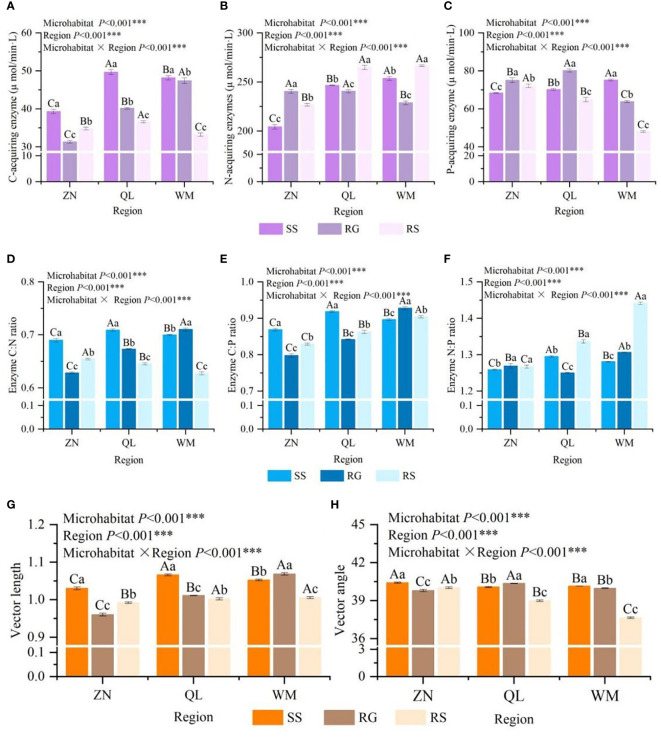
Characteristics of soil extracellular enzyme activities in different karst microhabitats **(A–C)**, soil extracellular enzyme stoichiometric characteristics among different karst microhabitats **(D–F)**, and vector characteristics of soil extracellular enzyme among different karst microhabitats **(G, H)**. Different lowercase letters on the column indicate significant differences between different karst microhabitats in the same region, and different uppercase letters indicate significant differences between different regions of the same karst microhabitats (p < 0.05).

There were significant differences in C- acquiring enzyme (CAE), N- acquiring enzyme(NAE) and P-acquiring enzyme(PAE) activities among different karst microhabitats (*p<* 0.05) (**Figures 1A–C**), but the changes were inconsistent. The N- and P-acquiring enzyme activities for ZN and P-acquiring enzyme activities for QL were RG>RS>SS, the N-acquiring enzyme activities for QL and WM were RS>SS>RG, the activities of C-acquiring enzyme activities and WM were the highest in SS. By comparing the differences of the same karst microhabitat among different regions, it was found that the enzyme activities of SS, RG and RS among different regions were significantly different (*p*< 0.05), indicating that the soil in karst area also had high heterogeneity among different regions. The results of two-way analysis of variance showed that both the microhabitat and the region had significant effects on the activities of C-, N- and P-acquiring enzyme activities (*p*< 0.001), and there was also a significant interaction between the two on the activities of these enzymes (*p*< 0.001).

### 3.3 Extracellular enzyme activity in different karst microhabitats

**Figure 2:**
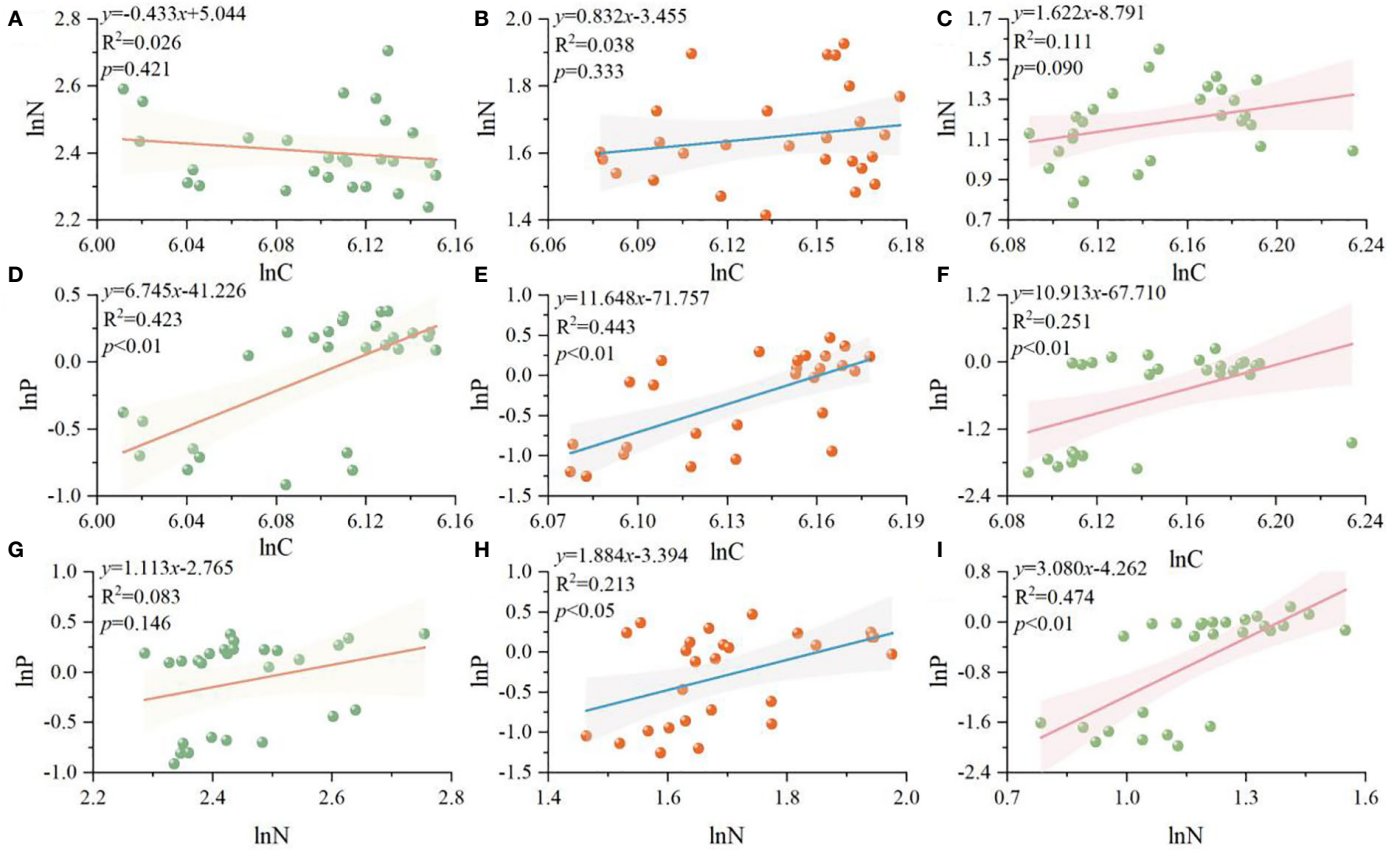
C, N, P standardized major axis regression analysis (SMA) of different organs of R. pudingense. Leaf **(A, D, G)**; stem **(B, E, H)**; root **(C, F, I)**.

Other than enzyme N:P showing no significant difference in ZN between RG and RS (*p* > 0.05), there were significant differences found in enzyme stoichiometric ratios among both karst microhabitats and regions (*p*< 0.05) (**Figures 2D–F**). For enzymes C:N and C:P, ZN and QL were the highest in SS, while WM was the highest in RG. The vector angles were significantly different in different regions and different karst microhabitats (*p* > 0.05). The vector characteristics of soil extracellular enzymes intuitively reflected the nutrient limitation of soil microbial metabolism among different karst microhabitats (**Figures 1G, H**). The vector length results showed that SS in the ZN and QL regions were most severely limited by carbon, while RG was most severe in the WM region. The vector angle of soil extracellular enzyme activities in three karst microhabitats was all less than 45°, and there were significant differences among microhabitats and regions (*p*< 0.05), indicating that soil microbes in the study area were subject to varying degrees of nitrogen limitation. Two-factor analysis of variance showed that different regions and different karst microhabitats had extremely significant effects on enzyme C:N, C:P, N:P, vector length, and vector angle (*p*< 0.001), and regions and microhabitats have an extremely significant interaction effect on enzyme stoichiometry ratios (*p*< 0.001).

### 3.4 Nutrient and stoichiometric characteristics of different organs

According to **Table 2**, there were significant differences (*p<* 0.05) in N content and C:N among different organs of *R. pudingense*, and leaf N content in RS significantly higher than that in SS and RG (*p<* 0.05), and leaf C content in RG and RS significantly lower than root C content (*p<* 0.05). Nevertheless, no significant differences (*p* > 0.05) were observed in P content, C:P, and N:P among different karst microhabitats and organs. The allocation pattern of N and P content in different organs showed a trend of leaf > stem > root, while C content showed the opposite trend. Moreover, the C:N and C:P in different organs revealed a pattern of root > stem > leaf, whereas N:P showed an entirely opposite trend. From the perspective of microhabitats, *R. pudingense* growing on the RS exhibited higher N and P contents but lower C content in leaves and stems than that growing on SS. Moreover, roots exhibited higher N content but lower C and P contents, resulting in lower C:N and higher C:P and N:P values. In addition, the C content was highest in all organs of plants growing on SS, whereas P content was higher in the roots. The indicators in RG were at a moderate level. The results of **Table 3** demonstrate that different organs had significant effects on nutrient allocation and stoichiometry of C, N, and P (*p<* 0.01); microhabitats significantly affected the N content and C:N ratio in plant organs (*p<* 0.05); N:P was influenced significantly by the interaction of microhabitats and organs (*p*< 0.05).

**Table 2:** Contents and stoichiometric ratios of C, N and P in different organs of *R. pudingense* among different karst microhabitats.

Organ	Microhabitat	C	N	P	C:N	C:P	N:P
Leaf	SS	451 ± 13.4Aa	10.8 ± 1.05Ab	1.00 ± 0.41Aa	42.0 ± 3.66Ca	560 ± 296Aa	13.2 ± 6.65Aa
	RG	442 ± 19.0Ba	10.6 ± 0.78Ab	0.96 ± 0.38Aa	42.0 ± 4.27Ca	561 ± 287Aa	13.5 ± 7.00Aa
	RS	443 ± 23.0Ba	12.0 ± 1.69Aa	1.02 ± 0.33Aa	37.7 ± 5.92Ca	479 ± 165Aa	13.1 ± 5.13Aa
Stem	SS	471 ± 21.9Aa	5.21 ± 0.80Ba	0.84 ± 0.48Aa	90.2 ± 13.2Ba	762 ± 458Aa	8.47 ± 4.79Aa
	RG	469 ± 14.8Aa	5.41 ± 0.89Ba	0.92 ± 0.43Aa	88.2 ± 12.8Ba	692 ± 446Aa	7.50 ± 3.94Aa
	RS	464 ± 17.5Aba	5.11 ± 0.62Ba	0.82 ± 0.37Aa	90.8 ± 10.96Ba	698 ± 374Aa	7.78 ± 4.25Aa
Root	SS	461 ± 14.5Aa	3.06 ± 0.46Ca	0.66 ± 0.36Aa	157 ± 24.9Aa	1174 ± 986Aa	7.10 ± 5.51Aa
	RG	467 ± 11.8Aa	3.31 ± 0.80Ca	0.70 ± 0.41Aa	149 ± 31.6Aab	1239 ± 1122Aa	7.61 ± 5.71Aa
	RS	459 ± 18.1Aa	3.58 ± 0.44Ca	0.73 ± 0.44Aa	131 ± 12.3Ab	1242 ± 1168Aa	9.05 ± 7.97Aa

Different capital letters indicate that contents and stoichiometric ratios of C, N and P are significantly different between different organs (p < 0.05), and different lowercase letters indicate that contents and stoichiometric ratios of C, N and P are significantly different between different karst microhabitats (p < 0.05).

**Table 3:** Results of two-way ANOVA of microhabitat and organ on C, N, P content and stoichiometric ratio of *R. pudingense* (*F* value).

Factors	C	N	P	C:N	C:P	N:P
Microhabitat	0.794	2.171	0.034	2.051	0.012	0.041
Organ	12.096***	447.260***	3.695*	291.859***	7.032**	7.671**
Microhabitat × Organ	0.480	2.166	0.113	1.897	0.042	0.157

*p < 0.05; **p < 0.01; ***p < 0.001.

### 3.5 C, N, and P in different organs of *R. pudingense*

**Figure 3:**
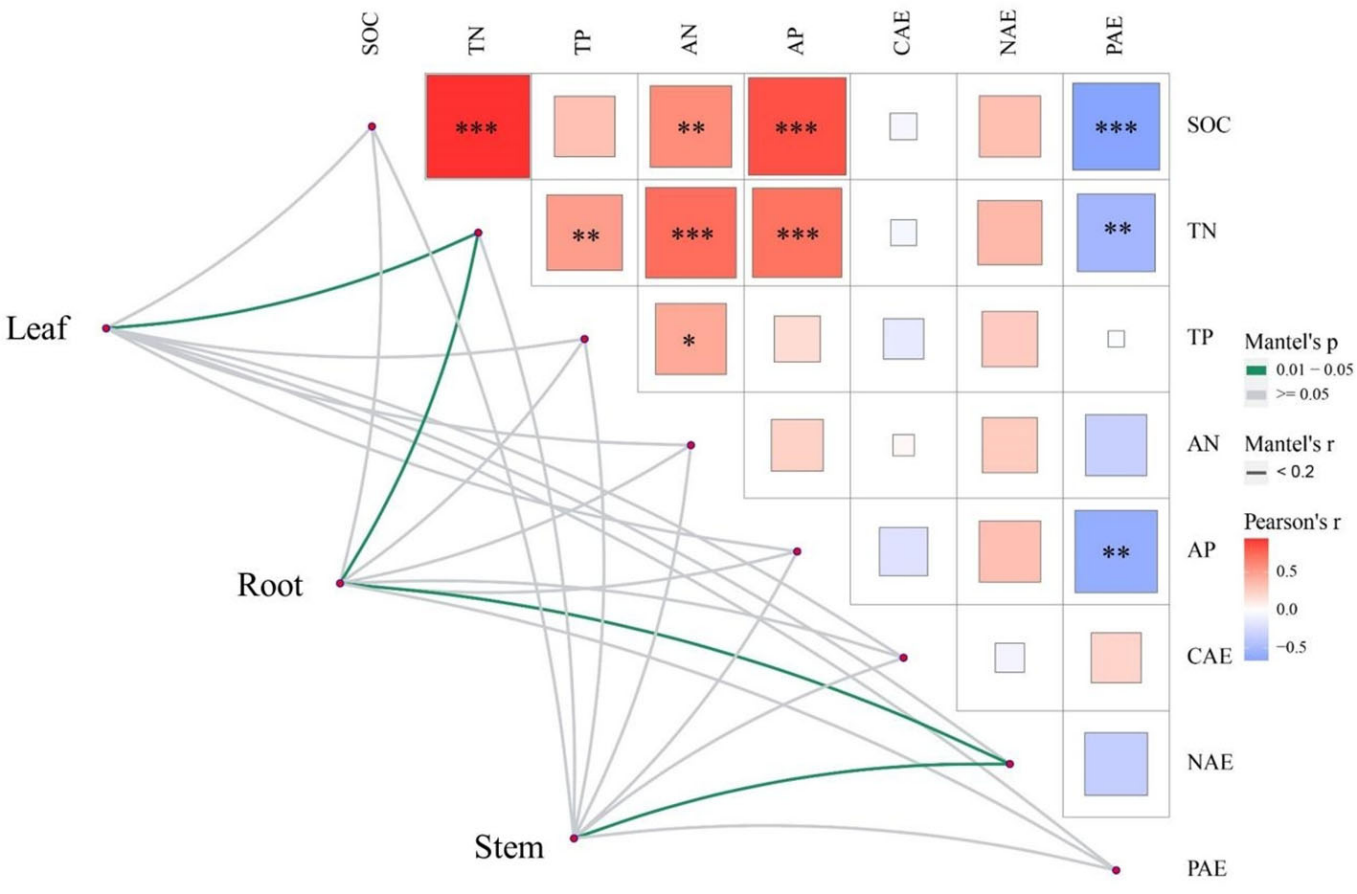
Correlation heat map of soil nutrients, enzyme activities and nutrient allocation in different organs of R. pudingense. The three maps correspond to three kinds of karst microhabitats. SOC, soil carbon content; TN, total nitrogen content; AN, available nitrogen content; TP, total phosphorus content; AP, soil phosphorus content; CAE, C- acquiring enzyme, NAE, N- acquiring enzyme; PAE, P- acquiring enzyme; ECN, enzyme C:N ratio; ECP, enzyme C:P ratio; ENP, enzyme N:P ratio. * p < 0.05; ** p < 0.01; *** p < 0.001.

Based on **Figures 2**, **3**, the allometric relationships of C, N, and P in different organs of *R. pudingense* exhibit various patterns. In leaves, there is a significant positive correlation between C-P (*p<* 0.01), and the slope is 6.745. Both C-P and N-P exhibit significant positive allometric relationships in stems (*p*< 0.05), and the slopes are 11.648 and 1.884 respectively. Similarly, the allometric relationships of C-P and N-P in roots are also significant and positive (*p*< 0.05), with slopes of 10.913 and 3.080, respectively.

### 3.6 Nutrient and stoichiometric characteristics of different organs

**Figure 4:**
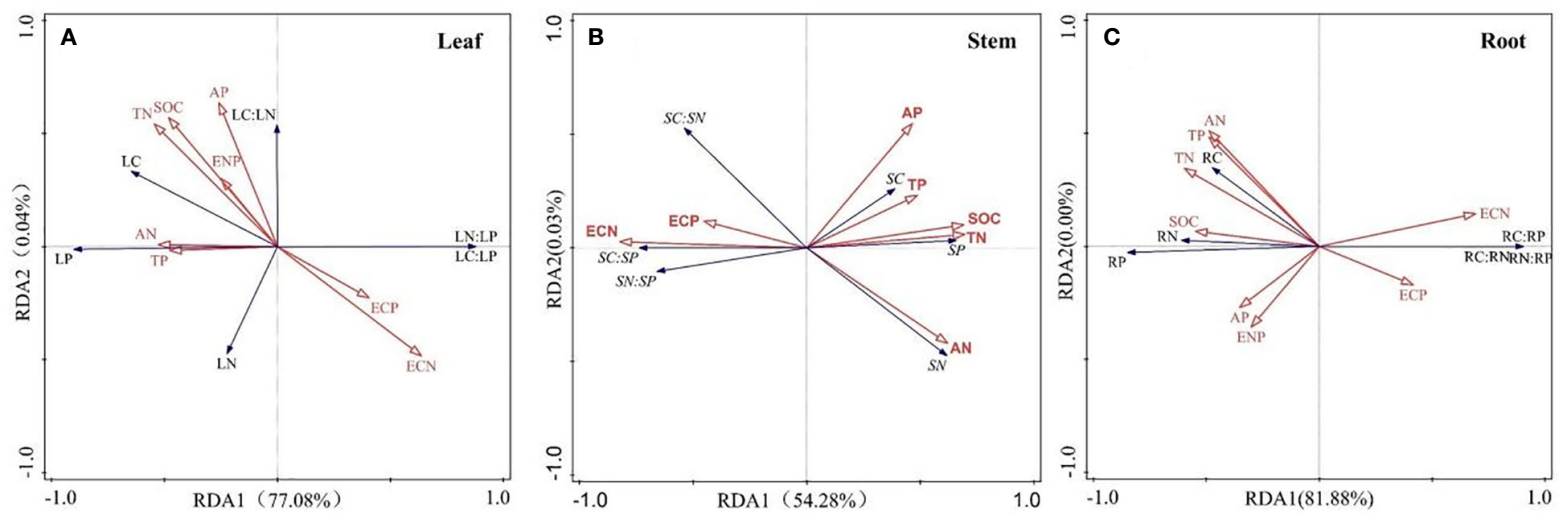
Redundancy analysis of environmental factors and stoichiometric ratios in different organs of *R. pudingense*. **(A)** Leaf; **(B)** Stem; **(C)** Root. LC, leaf carbon content; LN, leaf nitrogen content; LP, leaf phosphorus content. SC, stem carbon content; SN, stem nitrogen content; SP, stem phosphorus content. RC, root carbon content; RN, root nitrogen content; RP, root phosphorus content.

Pearson correlation analysis was conducted on 11 soil factors, and the results showed SOC was positively correlated with TN and AN (*p*< 0.05), TN was positively correlated with TP, AN and AP (*p*< 0.05) (**Figure 3**). Both SOC and TN were negatively correlated with AP (*p*< 0.01), and AP was negatively correlated with PAE (*p*< 0.01). The relationship between enzyme stoichiometry ratios was also closely related (*p*< 0.05). The results of the Mantel test indicated that different nutrient allocation strategies in different organs of *R. pudingense* were inconsistently affected by environmental factors. TN significantly affected the allocation of nutrients in the leaf and root (*p*< 0.05), while NAE was significantly correlated with the nutrient allocation of the stem and leaf (*p*< 0.05). RDA analysis revealed that for the leaf of *R. pudingense* (**Figure 4A**), soil factors explained 78.12% of the total variation, with the first two axes explaining 78.08% and 0.04%, respectively. ECN, TP, and ENP significantly affected leaf stoichiometric characteristics (*p*< 0.05). For the stem (**Figure 4B**), soil factors explained 54.31% of the total variation, with the first two axes explaining 54.28% and 0.03%, respectively. ECN and TP significantly affected the stoichiometric characteristics of the leaves (*p*< 0.05). For the roots (**Figure 4C**), soil factors explained 81.88% of the total variation, with ECN, TP, and ENP significantly affecting the stoichiometric characteristics of the leaves (*p*< 0.05).

## 4 Discussion

### 4.1 Soil nutrient and enzyme stoichiometry ratio analyses

It’s found that differences and interactions between microhabitat and region scales significantly affected the content and stoichiometry of soil C, N, and P (*p<* 0.05), with AN and AP content being more influenced by microhabitat (*p<* 0.05). This suggests that the high level of heterogeneity observed in karst ecosystems is a consistent feature across various microhabitats and regions. This has important implications for ecological research, as it underscores the need for careful consideration and evaluation of small-scale differences when investigating larger ecological patterns in karst regions. Consistent with previous research Liu et al. (2008), the nutrient content in SS was lower than that in RG and RS. This may be due to the differences in water and heat conditions among different microhabitats (Yu et al., 2011; Liao et al., 2013), which affect soil microbial activity (Gao et al., 2021). Additionally, the stability of soil aggregates was weakest in SS, causing more severe soil erosion during heavy rainfall and subsequent nutrient loss (Wei et al., 2022). The soil C, N, and P content in ZN were lower than those in QL and WM, possibly due to differences in tree density and biodiversity among regions (**Table 1**). The density of trees in the QL community is higher than that in ZN, and the region receives more precipitation. The temperature in WM is higher, affecting soil microbial activity and enzyme activity through temperature, humidity, and litter substrate factors (Álvarez-Yépiz et al., 2008), ultimately resulting in heterogeneity of soil nutrients in different microhabitats and regions. Due to the high heterogeneity of karst habitat and diverse microhabitat combinations, it was challenging to ensure complete consistency in microhabitat selection. Therefore, only the most typical SS, RG, and RS habitats of *R. pudingense* were selected. Additionally, there were differences in slope, rock exposure rate, and other factors even within the same microhabitat due to the unique structure of these habitats. Factors such as slope, soil thickness, and rock exposure rate have a certain impact on the soil environment (Wang L. et al., 2015; Peng et al., 2016) and small-scale climate (Jourgholami et al., 2019) of microhabitats, which led to significant variations in soil physicochemical properties in RG and RS in the results, further confirming the complexity and high heterogeneity of karst microhabitats.

At the global scale, the enzyme C:N:P ratio in soils is close to 1:1:1 (Sinsabaugh et al., 2008), but given the diversity of global ecosystem types and varying environmental conditions where plants grow, some researchers have found this equilibrium difficult to maintain (Guan et al., 2022). In this study, the soil enzyme C:N:P ratios, after logarithmic transformation, were calculated to be 1:1.49:1.15, which deviated from the expected values. N-acquiring enzyme activities were higher in karst microhabitats, and the enzyme vector angles were less than 45°, indicating relative nitrogen scarcity in the area. When microbes require a limited nutrient to meet metabolic demands, they typically secrete more specific enzymes, leading to these deviations (Sinsabaugh et al., 2008). Overall, there were significant differences in extracellular enzyme stoichiometry among different karst microhabitats (*p<* 0.05). Enzyme C:N ratios and vector lengths were highest in SS and lowest in RG and RS, while vector angles were the opposite, suggesting weaker nitrogen limitation in SS compared to RG and RS. The reason may be that the exposed bedrock in RG and RS causes greater temperature fluctuations (Yan et al., 2019), inhibiting microbial activity and reducing nitrogen mineralization. During the experimental design phase, we selected sampling points from three distinct regions while maintaining similar forest vegetation types within each region. The aim was to identify phenomena of differing nutrient limitation patterns (N *vs* P) that may exist across these regions, ultimately resulting in potentially divergent adaptive strategies. Interestingly, all regions were subjected to varying degrees of N limitation, which enables us to more precisely address the previously posed question of “how *R. pudingense* will allocate its nutrients under current nutrient limitations” by focusing on a single mode of limitation.

### 4.2 Nutrient allocation strategies of *R. pudingense*

Roots, stems, and leaves are critical organs for nutrient synthesis in plants, and the nutrient allocation pattern between these organs can reflect the plant’s ability to acquire, transport, and store nutrients (Chapin, 1980; Chapin et al., 1990). In this study, the content of C, N, and P in *R. pudingense* were 408.25-509.79 g/kg, 2.19-14.95 g/kg, and 0.14-1.59 g/kg, respectively. The C content was not only comparable to that of Chinese shrub leaves (449.1 g/kg) (Zhao et al., 2018), but also approached the global average for terrestrial plant leaves (464.00 g/kg) (Elser et al., 2000). However, the N and P content were lower than the global average (20.60 and 1.99 g/kg) (Elser et al., 2000). The C content was similar among different organs, while the content of P had no significance SOC content in the study area was significantly higher than that reported by Sun et al. (2022) in the karst rocky desertification ecosystem (22.23-35.60 g/kg). These findings suggest that *R. pudingense* has a relatively balanced utilization of C in an environment where C and P elements are not strongly limited or have little impact on the plant, while the limitation of N elements has a greater impact on this specie. Koerselman and Meuleman (1996) suggested that when the N:P ratio of plants falls below 14, this indicates that the plant is experiencing N limitation. Notably, all organs of R. *pudingense* exhibit N:P ratios that are lower than 14, a finding that we posit is indicative of habitat-driven N limitation.

The “growth rate hypothesis” proposes that rapid growth of organisms requires a large amount of ribosomal RNA synthesis for protein production, and because ribosomal RNA contains a large amount of P, high-growth organisms have low C:P and N:P ratios (Elser et al., 2000). In this study, the C:P ratio exhibited a root > stem > leaf pattern in *R. pudingense*, consistent with the findings of Liu et al. (2022), where leaves had the fastest growth rate and therefore the lowest C:P ratio. However, the N:P ratio in different organs of *R. pudingense* showed a completely opposite trend, possibly due to the plant’s strategy to cope with N-limited environments by prioritizing the allocation of more limited N to leaves (**Table S3**). N content determines the photosynthetic rate of plants and the synthesis of enzymes required to meet normal physiological demands (De Groot Corine et al., 2003). Litter decomposition is an important nutrient input into the karst forest ecosystem, and *R. pudingense* stores more N in its leaves, which, after falling, compensate for soil nutrients through extracellular enzyme hydrolysis. For plants, this is a very effective survival strategy. Under favorable conditions, nutrient allocation among organs is relatively even to ensure the growth intensity of each organ, while under strong N limitation, more energy and nutrients are allocated to aboveground parts to increase N use efficiency and ensure survival priority, forming a clear trade-off between different organs. This is the “adaptive growth hypothesis” proposed by Zhang et al. (2020) and helps us understand the adaptive strategies chosen by *R. pudingense* in N-limited environments. The C:N ratio of *R. pudingense* was higher than that of terrestrial plants globally (30.9 g/kg), indicating higher N use efficiency of *R. pudingense* in strong N-limited habitats.

The distribution of nutrients and stoichiometry ratios in *R. pudingense* is notably shaped by distinct organs, with only minor influence from microenvironments. This contradicts our previous beliefs. Nevertheless, it does not entirely negate the role of microenvironments in shaping nutrient allocation strategies employed by *R. pudingense*. Our findings suggest that various karst microenvironments encounter differing degrees of nitrogen limitation, and *R. pudingense* primarily adapts its nutrient allocation within its body to deal with nitrogen-limited conditions. These microenvironments might also impact the stability of soil aggregates (Wei et al., 2022), the microbiome community (Yuan et al., 2023), and other factors that alter the effectiveness of soil nutrients and thus affect the nutrient allocation of *R. pudingense*.

There may be some allometric relationships between nutrient contents in different plant organs (Liu et al., 2010). Apart from the negative correlation between leaf C and N, this study reveals positive correlations between C-N, C-P, and N-P contents across different organs of *R. pudingense* (**Figure 2**). The significant positive correlations (*p<* 0.05) in the C-P and N-P allometric models of root, stem, and leaf suggest similarity in the demand for C and P elements among plant organs. There is no significant allometric relationship between C and N in different organs (*p* > 0.05), possibly due to stronger N limitation in the environment (Yin et al., 2021). Compared with stems and roots, the slope of the leaf N-P allometric equation is smaller, indicating faster N accumulation and more stable N absorption. Under N deposition, challenges such as reduced C:N ratio in plants (Zhang et al., 2018), soil acidification, and restricted root growth (Liu et al., 2020) may arise, which require further analysis of *R. pudingense* functional traits to uncover its adaptation mechanisms in harsh karst environments.

### 4.3 Drivers of nutrient allocation strategies

*R. pudingense* adopts a nutrient allocation strategy of directing limited N resources primarily to photosynthetic organs such as leaves to cope with N limitation, which is consistent with some previous studies (Zhao et al., 2021). SOC content is an important indicator of soil nutrient availability for N and P, and studies have shown that the cycling rate of soil carbon is closely related to the effectiveness of N in ecosystems under N limitation (Singh et al., 2010; Yang et al., 2011). Dong et al. (2021) found that microbial activity was inhibited under N-limited conditions, leading to weakened SOC decomposition. In this study, SOC was significantly positively correlated with TN and AN, which is consistent with the aforementioned research conclusions. When the AP content increased, P-acquiring enzyme activities decreased significantly, following the predictions of the “resource allocation theory”. When AP content is abundant in the soil, microbes balance their own needs and invest more resources to obtain the limited elements, thereby reducing the quantity and activity of P-acquiring enzymes (Sinsabaugh et al., 2008). Mental test analysis showed that TN significantly influenced nutrient allocation to leaves and roots (*p<* 0.05), while AN significantly impacted nutrient allocation to roots and stems. This might be due to the fact that roots were the organs through which plants absorbed nutrients from the soil, the deficiency of N in karst microhabitats caused *R. pudingense*’s leaves to accumulate more N elements, leading to various organs of *R. pudingense* being relatively sensitive to indicators related to soil N elements. N-acquiring enzymes were closely related to the effectiveness of soil N and indirectly affect the nutrient transport rate to roots and stems (Ordoñez et al., 2009). Therefore, TN and NAE exert the greatest influence on the nutrient allocation of *R. pudingense*.

## 5 Conclusions

In this study, the complex interplay between microhabitat and regional scales significantly influences soil carbon, nitrogen, and phosphorus content in karst ecosystems, revealing pronounced heterogeneity. The deviation from the conventional 1:1:1 C:N:P ratio at a global scale underscores relative nitrogen scarcity in karst microhabitats, leading to altered enzyme stoichiometry. R. pudingense demonstrates a balanced utilization of carbon and phosphorus, while actively prioritizing nitrogen allocation in response to its N-limited habitat, reflecting an adaptive growth strategy. Microhabitat differences minimally impact nutrient allocation within the plant, but indirectly influence soil stability and microbiome communities. Allometric relationships between nutrient contents in different plant organs highlight the dynamic nature of nutrient allocation, with positive correlations between carbon-phosphorus and nitrogen-phosphorus contents. The drivers of nutrient allocation strategies in R. pudingense are shaped by factors such as soil organic carbon content, total nitrogen content, and N-acquiring enzyme activity, revealing how the plant adapts to nutrient limitations in karst microhabitats. Overall, this study provides comprehensive insights into the intricate ecological dynamics of karst regions, emphasizing the need for nuanced consideration of microhabitat variations in ecological research.

## Data availability statement

The raw data supporting the conclusions of this article will be made available by the authors, without undue reservation.

## Author contributions

HW: Conceptualization, Data curation, Investigation, Methodology, Writing – original draft. BH: Writing – review & editing. HZ: Writing – review & editing. XD: Writing – review & editing. MC: Writing – review & editing. FD: Writing – review & editing. PW: Writing – review & editing. LH: Writing – review & editing. RY: Conceptualization, Funding acquisition, Writing – review & editing. CY: Conceptualization, Data curation, Funding acquisition, Investigation, Writing – original draft, Writing – review & editing.
