# Supplementary material for: Unraveling plant adaptation to nitrogen limitation from enzyme stoichiometry aspect in Karst soils: a case study of Rhododendron Pudingense
Source: Front Plant Sci. 2023 Nov 30;14:1267759. doi: 10.3389/fpls.2023.1267759 (PMC10720638; doi:10.3389/fpls.2023.1267759)
Supplement: Supplementary file 1 [file DataSheet_1.docx]

Supplementary Information

Unraveling plant adaptation to nitrogen limitation from enzyme stoichiometry aspect in Karst soils: A case study of *Rhododendron Pudingense*

Haodong Wang ^1,2,3†^, Baoxian Huang ^1†^, Hongjiu Zhao ^1^, Xiaoyong Dai ^2^, Meng Chen ^2^, Fangjun Ding ^2,3^, Peng Wu ^2,3^, Lei Hao ^4^, Rui Yang ^1^*, Congjun Yuan ^2,3^*

^1^ College of Forestry, Guizhou University, Guiyang 550025, China

^2^ Guizhou Academy of Forestry, Guiyang 550005, China

^3^ National Positioning Observation and Research Station of Guizhou Libo Karst Forest Ecosystem, Libo, Guizhou 558400, China

^4^ Guizhou Provincial Institute of Biology, Guiyang, Guizhou 556000

***** Corresponding authors: yr553017@163.com

***** Co-corresponding authors: ycongjun2012gzdx@126.com

^†^ These authors contributed equally to this work.


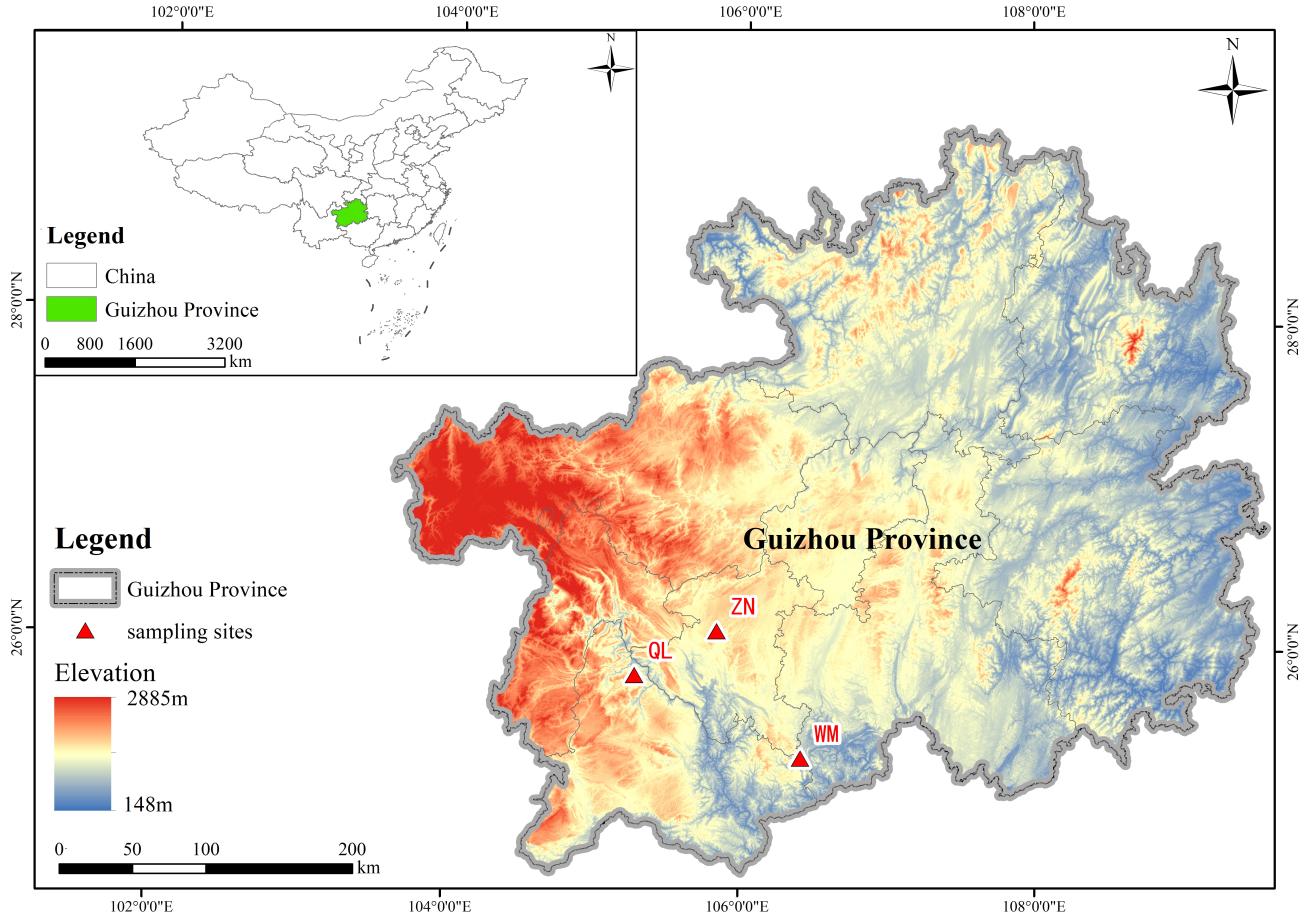


**Figure S1** Location and basic information of the study area and sampling sites (Reproduced from Yuan et al., 2023).

**Table S1** Characteristics of karst microhabitat.

| Microhabitat | Grown form | Soil characteristics |
| --- | --- | --- |
| Soil surface (SS) | The soil coverage area is greater than 1 m^2^ or, if the soil coverage area is less than 1 m^2^, then the bare rock rate is less than 50% and the soil is the dominant component, without the formation of rock potholes or groove depths exceeding 30 cm. | The soil layer on the ground surface is relatively thick, with good ventilation conditions and slow water loss. However, the soil aggregates have poor stability. |
| Rock gully (RG) | The exposed rate of bedrock is greater than 50%, and the covering area of continuous soil is less than 1 m^2^. The depth of the resulting rock groove is more than 30cm. Alternatively, even if the depth of the rock grooves or cavities formed is not more than 30cm, the thickness of the soil layer is over 30cm. Or, although the exposed proportion of bedrock is less than 50% and the predominant component is soil, the depth of the resulting rock groove or cavity is more than 30 cm. | The soil layer is thick, which is not prone to water loss, and has good water and fertility retention. Additionally, the soil aggregates have excellent stability. |
| Rock surface (RS) | The exposed rate of bedrock is greater than 50%, and the soil coverage area is less than 1m^2^ with rock groove depths not exceeding 30 cm, and the depth of the soil layer should not exceed 30 cm. | The ventilation conditions are good, which leads to quick water dispersal and weak water and fertility retention ability. As a result, it is prone to frequent temporary droughts in the short term. Furthermore, the stability of soil aggregates is relatively poor. |

**Table S2** Soil nutrient contents in different karst microhabitats (Cited from our previous work (Yuan et al., 2023)).

| **Reion** | **Microhabitat** | **SOC**  **(g/kg)** | **TN**  **(g/kg)** | **TP**  **(g/kg）** | **AN**  **(mg/kg)** | **AP (mg/kg)** |
| --- | --- | --- | --- | --- | --- | --- |
| ZN | SS | 97.23±41.91Aa | 8.54±3.45ABa | 0.77±0.20Aa | 448.52±142.86Aa | 30.20±15.38Aa |
|  | RG | 74.43±41.07Ba | 8.01±1.65Ba | 0.61±0.23Ba | 385.70±222.52Ba | 27.89±9.44Aa |
|  | RS | 94.77±28.45Ba | 8.12±1.84Ca | 0.61±0.12Ba | 580.41±29.11Aa | 24.08±10.79Ba |
| QL | SS | 137.90±11.33Aa | 13.76±2.74Aa | 0.72±0.10Ab | 750.99±64.70Aa | 22.02±1.43Aa |
|  | RG | 208.93±29.87Aa | 19.44±3.30Ab | 1.36±0.14Aa | 951.63±324.54Aa | 60.09±44.76Aa |
|  | RS | 134.88±33.88Ba | 14.12±1.74Bab | 1.16±0.26Aa | 761.58±67.23Aa | 25.45±6.65Ba |
| WM | SS | 75.30±13.29Ac | 5.65±0.92Bc | 0.59±0.19Aa | 378.07±24.79Ab | 36.24±4.05Ab |
|  | RG | 237.02±38.44Ab | 17.25±3.69Ab | 0.71±0.08Ba | 858.03±202.08ABab | 57.66±25.11Ab |
|  | RS | 338.30±58.49Aa | 23.07±1.68Aa | 0.92±0.07ABa | 950.52±535.62Aa | 143.46±71.89Aa |

Note: SOC, soil carbon content; TN, total nitrogen content; AN, available nitrogen content; TP, total phosphorus content; AP, soil phosphorus content. Different capital letters indicate significant differences between different regions at the same karst microhabitat (p < 0.05); different lower-case letters indicate significant differences between different karst microhabitats at the same region (p < 0.05). The same below.

**Table S3** Results of two-way ANOVA of microhabitat and region on soil nutrient contents (F value) (Cited from our previous work (Yuan et al., 2023)).

| Factors | SOC | TN | TP | AN | AP |
| --- | --- | --- | --- | --- | --- |
| Microhabitat | 29.028*** | 25.559*** | 15.955*** | 5.187* | 7.508** |
| Region | 14.700*** | 15.382*** | 4.226* | 2.637 | 2.968 |
| Microhabitat × Region | 15.560*** | 13.439*** | 5.584** | 1.761 | 4.485* |

Note: * p < 0.05, ** p < 0.01, *** p < 0.001. The same below.
